# Inter‐hospital transfer for thrombectomy: transfer time is brain

**DOI:** 10.1111/ene.16276

**Published:** 2024-03-14

**Authors:** Pierre Seners, Maeva Khyheng, Julien Labreuche, Bertrand Lapergue, Fernando Pico, the ETIS investigators, Bertrand Lapergue, Adrien Wang, Arturo Consoli, Oguzhan Coskun, Federico Di Maria, Silvia Pizzuto, Alessandro Sgreccia, Charline Benoit, Lucas Gorza, David Weisenburger‐Lile, Waliyde Jabeur, Maia Tchikviladze, Serge Evrard, Georges Rodesch, Raphaël Blanc, Michel Obadia, Jean‐Philippe Desilles, Michel Piotin, Pierre Seners, Stanislas Smajda, Simon Escalard, Benjamin Maier, Candice Sabben, Hocine Redjem, Mikael Mazhigi, Grace Adwane, François Delvoye, Amira Al Raaisi, William Boisseau, Adnan Altayeb, Erwan Robichon, Igor Raynouard, Cristina Hobeanu, Omer Eker, Tae‐Hee Cho, Laurent Derex, Julia Fontaine, Laura Mechtouff, Norbert Nighoghossian, ONG Elodie, Lucie Rascle, Roberto Riva, Françis Turjman, Morgane Laubacher, Mehdi Beyragued, Yves Berthezene, Marc Hermier, Ameli Roxanna, Alexandre Bani‐Sadr, Andrea Filip, Matteo Cappucci, Pierre Pernot, Benjamin Daumas Duport, Pierre Louis Alexandre, Hubert Desal, Solene De Gaalon, Benoît Guillon, Cécile Preterre, Guillaume Tessier, Arthur Lionnet, Benjamin Gory, Lisa Humbertjean‐Selton, René Anxionnat, Anne‐Laure Derelle, Liang Liao, Emmanuelle Schmitt, Sophie Planel, Sébastien Richard, Gioia Mione, Jean‐Christophe Lacour, Marian Douarinou, Emilien Micard, Bailiang Chen, Gérard Audibert, Agnès Masson, ALB Lionel, Marine Beaumont, Adriana Tabarna, Marcela Voicu, Grégoire Barthel, Iona Podar, Madalina Brezeanu, Marie Reitter, François Zhu, René Anxionnat, Gaultier Marnat, Jean‐Sébastien Liegey, Pierre Briau, Victoire Lyon, Igor Sibon, Xavier Barreau, Jean Papaxanthos, Jerome Berge, Sabrina Debruxelles, Stéphane Olindo, Mathilde Poli, Pauline Renou, Sharmila Sagnier, Thomas Tourdias, Thomas Courret, Malgorzata Milnerowicz, François Rouanet, Benoit Legghe, Aurelie Boyer, Tristan Kerdraon, Lisa Saleille, Laurine Bastardo, Cyril Dargazanli, Vincent Costalat, Federico Cagnazzo, Pierre‐Henri Lefevre, Grégory Gascou, Quentin Varnier, François Louis Collemiche, Isabelle Mourand, Caroline Arquizan, Lucas Corti, Adrien Ter Schiphorst, Imad Derraz, Laurent Spelle, Jildaz Caroff, Christian Denier, Vanessa Chalumeau, Cristian Mihalea, Nicolas Legris, Augustin Ozanne, Leon Ikka, Olivier Chassin, Sophie Gallas, Laura Venditti, Mariana Sarov, Jonathan Cortese, Jean‐Christophe Ferre, Stephane Vannier, Maria Veronica Lassalle, Jean‐Yves Gauvrit, Clément Tracol, Cécile Malrain, Edouard Beaufreton, Quentin Alias, Julien Hissier, Maud Guillen, François Eugene, Julien Boucherit, Cyril Chivot, Audrey Courselle, Sandrine Canaple, Chantal Lamy, Kevin Delaforge, Manuel Fernandez, Jérémie Vial, Quentin Laferte, Xavier Desdoit, Serge Timsit, Aurore Jourdain, Jean‐Christophe Gentric, Julien Ognard, Mourad Cheddad El Aouni, Irina Viakhireva, Jordan Coris, François‐Mathias Merrien, Denis Marechal, Marie Bruguet, Philippe Goas, Marion Boulanger, Emmanuel Touze, Charlotte Barbier, Romain Schneckenburger, Julien Cogez, Sophie Guettier, Estelle La Porte, Jean Bouchart, Mohammad Ismail, Charbel Mounayer, Aymeric Rouchaud, Suzana Saleme, Géraud Forestier, Sonia Alamowitch, Charlotte Rosso, Flore Baronnet, Sophie Crozier, Anne Leger, Laure Bottin, Sam Ghazanfari, Marion Yger, Stephen Delorme, Aymeric Wittwer, Christine Vassilev, Gaspard Gerschenfeld, Clémence Blanc, Antonio Rainone, Frédéric Clarençon, Kevin Premat, Eimad Shotar, Stéphanie Lenck, Nader Sourour, Julien Allard, Pierre Marie Chiaroni, Olivier Naggara, Guillaume Turc, Wagih Ben Hassen, Denis Trystram, Christine Rodriguez‐Regent, Laza‐Nomenjanahary Finoana, Hugo Gortais, Ozlem Ozkul‐Wermester, Chrysanthi Papagiannaki, Evelyne Massardier, Aude Triquenot, Margaux Lefebvre, Julien Burel, Florian Basille, Adelya Curado, Alain Viguier, Christophe Cognard, Anne Christine Januel, Jean‐François Albucher, Lionel Calviere, Jean‐Marc Olivot, Nicolas Raposo, Fabrice Bonneville, Guillaume Bellanger, Louis Fontaine, Philippe Tall, Federico Sacchetti, Frédéric Bourdain, Patricia Bernady, Guillaume Ballan, Stephanie Bannier, Emmanuel Ellie, Olivier Flabeau, Julia Potenza, Antoine Soulages, Laurent Lagoarde‐Segot, Hélène Cailliez, Louis Veunac, David Higue, Quentin Bourgeois‐Beauvais, Anthony Lebras, Sarah Adam, Benoit Pegat, Arnaud Le Guen, François Chedeville, Jérémy Jouan, Rémi Allibert, Alexandre Courtois, Lucile Lescot, Dorothée Videt, Thibaut Lapotre, Johann Sebastian Richter, Bruno Thierry Barroso, Camille Dahan, Alexis Gonnet, Régis Hubrecht, Zoé Lepine, Hélène Castagnet, Raluca Marasescu, Olivier Heck, Pauline Cuisenier, Olivier Detante, Isabelle Favre Wiki, Clémentine Bonaz, Katia Garambois, Loic Legris, Adrian Kastler, Kamel Boubagra, Corentin Berthet, Stéphane Charara, Florent Lepilliet, Jérémie Papassin, François Lun, Carole Henry, Laurence Berthon, Mélanie Roussel, Richard Macrez, Serkan Cakmak, Victor Dumas, Jean Philippe Neau, Nathalie Nasr, Valérie Wolff, Raoul Pop, Véronique Quenardelle, Valérie Lauer, Irène Pierre‐Paul, Roxana Gheoca, Leonard Lorant, Solène Moulin, Vi Tuan Hua, Paolo Pagano, Alexandre Doucet, Christophe Gelmini, Pierre‐François Manceau, Laurenti Paiusan, Isabelle Serre, Sébastien Soize, Thi Ngoc Phuong Nguyen, Maher Sahnoun, Nathalie Caucheteux, Pablo Lebedinsky, Federico Bolognini, Francis Vuillemet, Anna Ferrier, Abderrahim Zerroug, Ricardo Moreno, Emmanuel Chabert, Elie Lteif, Pauline Paris, Nathalie Bourgois, Marie Raquin, Anne Pasco‐Papon, Jean Baptiste Girot, Alderic Lecluse, Sophie Godard, Vincent L’allinec, Alice Corfu, Kevin Janot, Richard Bibi, Marie Gaudron, Arnaud Bretonniere, Mariam Annan, Héloïse Ifergan, Grégoire Boulouis, Marco Pasi, Séverine Debiais, Elisabeth Molinier, Denis Herbreteau, Fouzi Bala, Clémence Hoche, Anthony Wietrich, Valérie Ruche, Karine Lavandier, Hakim Amara, Yannick Bejot, Brivael Lemogne, Frédéric Ricolfi, Laura Baptiste, Pierre Thouant, Gaulthier Duloquin, Pierre‐Olivier Comby, Christelle Blanc‐Labarre, Simon Amaral, Angélique Bernard, Adrien Chavent, Alan Rhamani, Corinne Chevalier, Guillaume Charbonnier, Louise Bonnet, Nicolas Raybaud, Benjamin Bouamra, Thierry Moulin, Alessandra Biondi, Fernando Pico, Benjamin Dano, Carmen Patcas, Dhouha Chaari, Daniela Stanciu, Duc Long Duong, Intissar Hmida, Marie‐Laure Chadenat, Laurent Suissa, Emilie Doche, Jean‐François Hak, Anthony Reyre, Xavier Carle, Nadia Laksiri, Ophélie Osman, Roxane Peres, Emmanuelle Robinet‐Borgmano, Cécile Dulau, Basile Kerleroux, Caroline Rey, Hervé Brunel, Philippe Dory‐Lautrec, Jean‐François Hak, Pierre Lehmann, Sivadji Vingadalassalom, Basile Kerleroux, Jérôme Berge, Federico Sacchetti, Fabrice Bing, Mathieu Bonnerot, Isabelle Berger, Nathalie Morel, Gilles Rodier, Wilfried Vadot, Barbara Casolla, Paul Clottes

**Affiliations:** ^1^ Neurology Department Rothschild Foundation Hospital Paris France; ^2^ Institut de Psychiatrie et Neurosciences de Paris (IPNP), UMR_S1266, INSERM, Université de Paris Paris France; ^3^ Biostatistics Lille University Hospital Lille France; ^4^ Neurology Department Foch Hospital Suresnes France; ^5^ Neurology Department Mignot Hospital Versailles France

**Keywords:** inter‐hospital transfer, ischaemic stroke, thrombectomy

## Abstract

**Background and purpose:**

Patients with acute ischaemic stroke and a large vessel occlusion who present to a non‐endovascular‐capable centre often require inter‐hospital transfer for thrombectomy. Whether the inter‐hospital transfer time is associated with 3‐month functional outcome is poorly known.

**Methods:**

Acute stroke patients enrolled between January 2015 and December 2022 in the prospective French multicentre Endovascular Treatment of Ischaemic Stroke registry were retrospectively analysed. Patients with an anterior circulation large vessel occlusion transferred from a non‐endovascular to a comprehensive stroke centre for thrombectomy were eligible. Inter‐hospital transfer time was defined as the time between imaging in the referring hospital and groin puncture for thrombectomy. The relationship between transfer time and favourable 3‐month functional outcome (modified Rankin Scale 0–2) was assessed through a mixed logistic regression model adjusting for centre and symptom‐onset‐to‐referring‐hospital imaging time, age, sex, diabetes, referring hospital National Institutes of Health Stroke Scale score, Alberta Stroke Programme Early Computed Tomography Score, occlusion site and intravenous thrombolysis use.

**Results:**

Overall, 3769 patients were included (median inter‐hospital transfer time 161 min, interquartile range 128–195; 46% with favourable outcome). A longer transfer time was independently associated with lower rates of favourable outcome (*p* < 0.001). Compared to patients with transfer time below 120 min, there was a 15% reduction in the odds of achieving favourable outcome for transfer times between 120 and 180 min (adjusted odds ratio 0.85; 95% confidence interval 0.67–1.07), and a 36% reduction for transfer times beyond 180 min (adjusted odds ratio 0.64; 95% confidence interval 0.50–0.81).

**Conclusions:**

A shorter inter‐hospital transfer time is strongly associated with favourable 3‐month functional outcome. A speedier inter‐hospital transfer is of critical importance to improve outcome.

## Graphical abstract

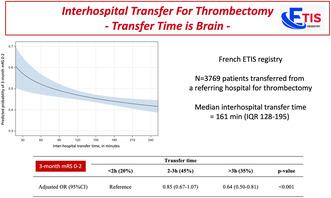

## INTRODUCTION

In patients with acute ischaemic stroke harbouring a large vessel occlusion (LVO), the efficacy of endovascular therapy (EVT) is well established in a large proportion of patients up to 24 h from symptom onset. Most LVO‐related stroke patients are first evaluated at primary stroke centres or community hospitals before being transferred to a centre that performs EVT [1]. The inter‐hospital transfer is a complicated process, involving numerous stakeholders and requiring a high degree of coordination between the referring hospital, transportation and receiving hospital teams. Hence, long delays are commonly observed, with median transfer times consistently exceeding 2–3 h in most settings [1, 2, 3]. The effectiveness of EVT is well known to be time‐sensitive, due to the progression of the irreversibly injured ischaemic brain tissue over time [4, 5]. However, this association has mainly been reported and quantified for elapsing time from symptom onset to EVT reperfusion, not specifically for inter‐hospital transfer time. Demonstrating such an association is of major importance, as it may underscore the urgent need for strategies aimed at expediting transfers, as well as indicate that inter‐hospital transfer is a promising time‐window for neuroprotection trials.

Here, the aim was to study whether inter‐hospital transfer time is associated with 3‐month functional outcome in a large multicentre registry.

## METHODS

Our analysis was conducted according to the STROBE criteria for observational studies. Informed consent was obtained from each patient or their relatives. The data supporting the study findings are available from the authors upon reasonable request.

### Data sources

The data were extracted from the Endovascular Treatment of Ischaemic Stroke (ETIS) registry between January 2015 and December 2022, for which detailed methods have been published previously. Briefly, ETIS is an ongoing French multicentre prospective observational study collecting data of consecutive patients with LVO undergoing EVT (NCT03776877). Data from acute stroke patients who fulfilled the following criteria were extracted for the current study: (1) initial admission at a non‐EVT‐capable centre where a brain imaging showed an anterior circulation LVO, (2) subsequent transfer to a comprehensive centre for consideration of EVT and (3) pre‐stroke modified Rankin Scale (mRS) score 0 or 1. All patients receiving groin puncture for EVT were included, regardless of whether EVT was eventually attempted and whether successful reperfusion was achieved.

### Clinical and radiological data

Clinical and imaging data routinely recorded in the acute stroke setting were collected. Imaging data were locally assessed by senior neuroradiologists. Time of inter‐hospital transfer was operationally defined as the time between baseline imaging in the referring hospital (i.e., when the LVO was first identified) and the groin puncture for EVT in the comprehensive centre. The primary outcome was favourable 3‐month outcome, defined as an mRS ≤2. Secondary outcome was the overall distribution of 3‐month mRS. Three‐month mRS scores were collected by certified investigators during routinely scheduled visits or by trained research nurses during a standardized telephone interview.

### Statistical analysis

The association of inter‐hospital transfer time with favourable outcome and overall mRS distribution (shift analysis) was assessed using mixed logistic regression models (binary for favourable outcome and ordinal for shift analysis) considering centre as a random effect and adjusted on the following pre‐specified confounding factors: symptom‐onset‐to‐referring‐hospital imaging time, age, sex, diabetes, admission (referring hospital) National Institutes of Health Stroke Scale (NIHSS) score, Alberta Stroke Programme Early Computed Tomography Score (ASPECTS), occlusion site and intravenous thrombolysis use. Finally, heterogeneity in associations between transfer time and favourable outcome according to symptom‐onset‐to‐referring‐hospital imaging time, NIHSS score, ASPECTS, occlusion site, intravenous thrombolysis use and time‐of‐day of referring hospital imaging was assessed by including the corresponding interaction term in the multivariable mixed logistic regression models [1, 6]. More details regarding statistical analysis are provided in Appendix S1.

## RESULTS

During the study period, 3769 patients met inclusion criteria. Mean (standard deviation) age was 70 (15) years and 1892 (50%) patients were male. Median (interquartile range, IQR) NIHSS score on admission in the referring hospital was 16 (IQR 10–20), and median time from symptom onset to referring hospital imaging was 127 min (IQR 97–174 min). On the referring hospital imaging, median ASPECTS was 8 (IQR 6–9) and occlusion site was the intracranial internal carotid in 596 (16%) patients and the first and second segments of the middle cerebral artery in 2597 (69%) and 573 (15%) patients, respectively. Intravenous thrombolysis was administered in the referring hospital in 2227 (59%) patients. Median inter‐hospital transfer time was 161 min (IQR 128–195 min). Transfer time was shorter than 120 min in 738 (20%) patients, between 120 and 180 min in 1705 (45%) patients and longer than 180 min in 1326 (35%) patients. Upon comprehensive centre arrival, the EVT procedure was attempted in 89.6% of patients, and successful reperfusion (modified Thrombolysis in Cerebral Infarction score 2b–3) was achieved in 89.1% of them. Median time from groin puncture to successful recanalization was 35 min (IQR 24–54 min) amongst patients successfully treated by EVT. Favourable outcome was observed in 1722 (46%) patients.

**FIGURE 1:**
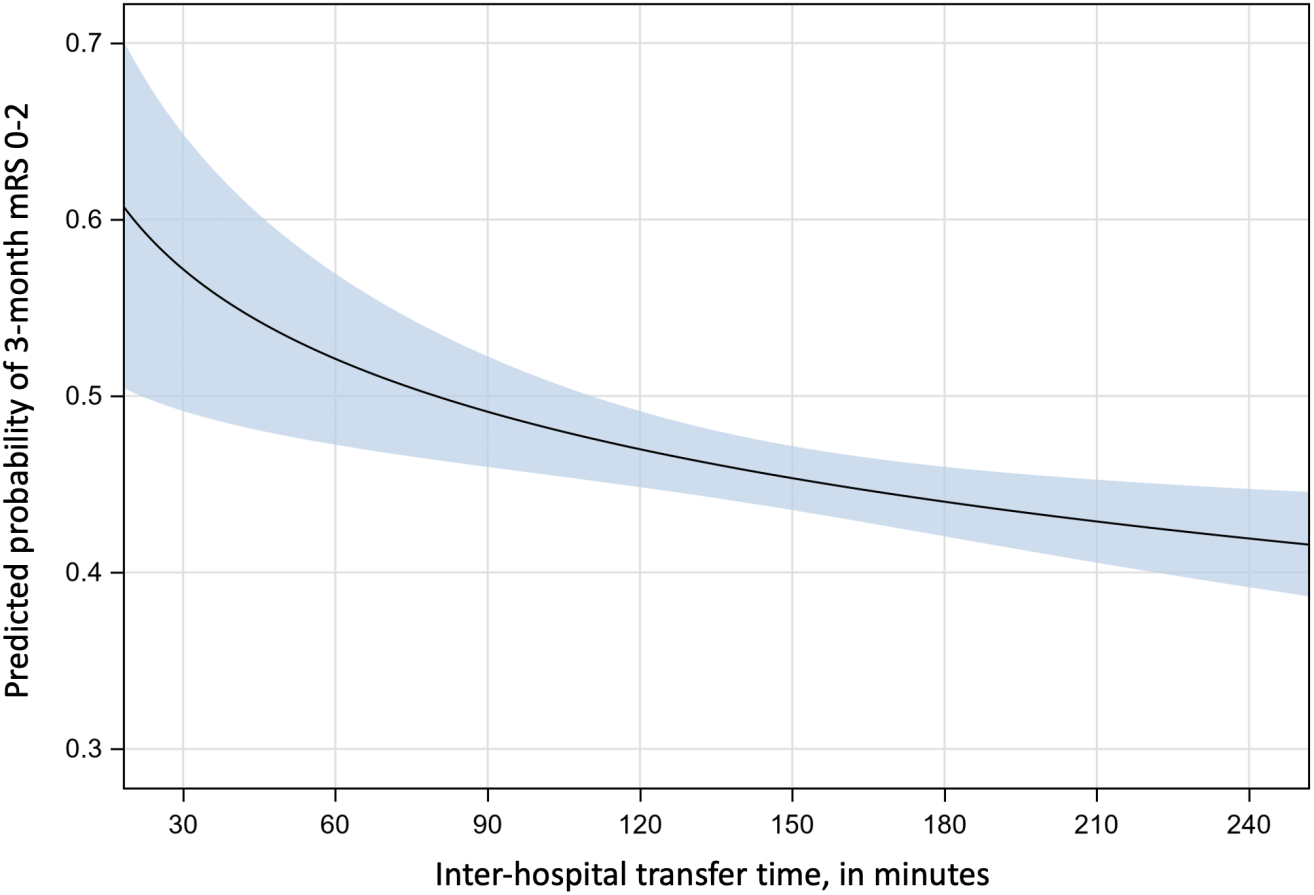
Association between inter‐hospital transfer time and favourable outcome. The regression curve estimates the probability of favourable outcome according to inter‐hospital transfer time for average patients. The shaded area corresponds to the 95% confidence interval (logistic regression model).

Longer inter‐hospital transfer time was independently associated with lower rates of favourable outcome (*p* < 0.001, Table 1 and Figure 1). Compared to patients with transfer time below 120 min, there was a 15% reduction in the odds of achieving favourable outcome for transfer times between 120 and 180 min (adjusted odds ratio 0.85; 95% confidence interval 0.67–1.07) and a 36% reduction for transfer times beyond 180 min (adjusted odds ratio 0.64; 95% confidence interval 0.50–0.81) (Table 1). Similar results were observed in the shift analysis across the entire mRS score (Table 1).

**TABLE 1:** Association between inter‐hospital transfer time and 3‐month functional outcome.

	Transfer time, as three‐level categorical variable				OR (95% CI)^a^	*p* value^a^
	<2 h	2–3 h	>3 h	*p* value^c^		
Favourable outcome (mRS 0–2)						
*N* (%)	363/738 (49.2)	805/1705 (47.2)	552/1326 (41.6)			
Centre adjusted OR (95% CI)	Reference	0.93 (0.76–1.13)	0.75 (0.60–0.93)	0.017	0.90 (0.83–0.98)	0.016
Fully adjusted OR (95% CI)^b^	Reference	0.85 (0.67–1.07)	0.64 (0.50–0.81)	<0.001	0.82 (0.74–0.90)	<0.001
Shift mRS						
Centre adjusted cOR (95% CI)	Reference	0.90 (0.76–1.07)	0.74 (0.60–0.90)	0.008	0.90 (0.84–0.97)	0.006
Fully adjusted cOR (95% CI)^b^	Reference	0.83 (0.69–1.01)	0.65 (0.53–0.80)	<0.001	0.83 (0.77–0.90)	<0.001

Abbreviations: CI, confidence interval; cOR, common OR; mRS, modified Rankin Scale; OR, odds ratio.

^a^ Odds ratio per 1 standard deviation log increase in transfer time and *p* values obtained using a mixed logistic regression model including log‐transformed transfer time as continuous variable.

^b^ Adjusted on centre, symptom‐onset‐to‐referring‐hospital imaging, age, sex, diabetes, admission (referring hospital) NIHSS score and ASPECTS, occlusion site and intravenous thrombolysis.

^c^ *p* values obtained using a mixed logistic regression model including transfer time as categorical variable.

No significant heterogeneity in the association between transfer time and favourable outcome was observed across the pre‐specified onset‐to‐referring‐hospital imaging time, admission (referring hospital) NIHSS score and ASPECTS, occlusion site, intravenous thrombolysis and time‐of‐day of referring hospital imaging subgroups (Figure S1).

## DISCUSSION

In this study including 3769 LVO‐related stroke patients transferred from a non‐endovascular centre for EVT, longer inter‐hospital transfer time was independently associated with poorer 3‐month functional outcome. To our knowledge, such association has never been shown. One study has reported a non‐significant trend between door‐in door‐out time in referring hospital and 3‐month outcome yet was limited by a small sample size [7].

Our data expand the known association between symptom onset to reperfusion time and functional outcome [4, 5] and highlight the urgent need for developing strategies to expedite inter‐hospital transfer to improve functional outcome.

Our results show that transfer time is long in day‐to‐day practice, with three‐quarters of patients with transfer time longer than 2 h, in line with other reports [2, 3]. There are several potential ways to speed up inter‐hospital transfer workflow, the first of which is reducing door‐in door‐out time in the referring hospital, with the establishment of in‐house protocols for early arterial imaging, cloud‐based image sharing with the EVT‐capable centre, and early mobilization of transport resources [8]. The use of positive feedback strategies has also shown benefit to reduce in‐hospital workflow [9, 10]. Quality improvement programmes focused on door‐in door‐out time should be used in non‐EVT‐capable centres to provide feedback on performance and improve workflow. The second way is shortening the transportation time, for instance facilitating the use of air transportation rather than ground ambulances in appropriate settings [11]. The third way is reducing door‐to‐reperfusion time upon comprehensive centre arrival, for example through direct admission in the angiosuite [12]. Last, transferring a flying intervention team instead of transferring the patient has been shown to significantly reduce time to EVT [13, 14].

In addition to transfer time reduction, which is of critical importance yet may reach a limit given the intricate nature of the process, innovative neuroprotective therapies aimed at slowing down infarct growth emerge as particularly relevant during inter‐hospital transfer [1, 2, 15]. Indeed, the potential benefits of neuroprotective therapies are more likely to be observed in transferred patients, where a substantial decrease in infarct growth could be achieved due to the expected long treatment exposure. This contrasts with patients directly admitted to endovascular‐capable centres, where shorter imaging‐to‐recanalization times are typically observed.

No significant heterogeneity in the association between transfer time and favourable outcome across our pre‐specified subgroups was observed, suggesting that fast transfer should be targeted regardless of baseline clinical or imaging characteristics. Poor leptomeningeal collaterals on computed tomography angiography or perfusion imaging have been shown as the primary factor associated with fast infarct growth during inter‐hospital transfer for EVT and may therefore help to identify a subgroup of patients for whom ultra‐fast inter‐hospital transfer should be prioritized [1, 2, 3]. However, collateral assessment in the referring hospital was not available in our registry. This warrants further research.

This study has limitations. The transferred patients who did not undergo groin puncture were not enrolled in our registry, which might have biased the results. However, a direct‐to‐angiosuite protocol is performed for transferred patients in most comprehensive stroke centres in France. Also, collateral or core volume assessment in the referring hospital was not recorded in our registry, impairing the study of heterogeneity according to these key variables.

## CONCLUSION

In LVO‐related acute stroke patients transferred from a non‐endovascular‐capable centre for EVT, longer inter‐hospital transfer time is strongly associated with poorer 3‐month functional outcome. Expediting inter‐hospital transfer is of critical importance to improve outcome.

## AUTHOR CONTRIBUTIONS

**Pierre Seners:** Conceptualization; writing – original draft; investigation; methodology; validation. **Maeva Khyheng:** Investigation; methodology; validation; writing – review and editing; formal analysis. **Julien Labreuche:** Investigation; methodology; validation; writing – review and editing. **Bertrand Lapergue:** Data curation; investigation; writing – review and editing; validation; project administration. **Fernando Pico:** Conceptualization; investigation; writing – review and editing; validation; methodology; supervision.

## FUNDING INFORMATION

None.

## CONFLICT OF INTEREST STATEMENT

None.

## Supporting information


Appendix S1.

## Data Availability

The data that support the findings of this study are available from the corresponding author upon reasonable request.
