# Supplementary material for: Inter‐hospital transfer for thrombectomy: transfer time is brain
Source: Eur J Neurol. 2024 Mar 14;31(6):e16276. doi: 10.1111/ene.16276 (PMC11235793; doi:10.1111/ene.16276)
Supplement: Supplementary file 1 — Appendix S1. [file ENE-31-e16276-s001.docx]

**Supplemental Materials**

**Supplemental Statistical Methods**

Quantitative variables are expressed as means (standard deviation, SD) in the case of normal distribution or medians (interquartile range) otherwise. Categorical variables are expressed as numbers (percentage). Normality of distributions was assessed using histograms and the Shapiro-Wilk test. We assessed the association of inter-hospital transfer time with favorable outcome and overall mRS distribution (shift analysis) using mixed logistic regression models considering center as random effect and using a binomial distribution and a logit link function for favorable outcome (binary logistic regression model) or a multinomial distribution and a cumulative logit link function for the shift analysis (ordinal logistic regression model). The models were adjusted on the following pre-specified confounding factors: time from symptom onset-to-referring hospital imaging, age, diabetes, admission (referring hospital) NIHSS and ASPECT scores, admission (referring hospital) occlusion site, and intravenous thrombolysis use. Inter-hospital transfer time was firstly analyzed as a 3-level categorical variable (<2, 2-3, >3hours, to appreciate the shape of the associations) and then as a continuous variable following log-transformation due to high skewness of distribution. Odds ratios (OR) of favorable outcome and common OR (cOR) for overall distribution of mRS with theirs 95% confidence intervals (CIs) were calculated for the uppers categories relative to the lowest, or for one standard deviation increase in log transformed value. Finally, we assessed heterogeneity in associations of inter-hospital transfer time (treated as continuous variable) with favorable outcome, according to onset-to-referring hospital imaging time (<120 vs. ≥120min and <360 vs. ≥360min), admission (referring hospital) NIHSS score (<16 vs. ≥16), admission (referring hospital) ASPECT score (<6 vs. ≥6), admission occlusion site (ICA vs. M1 vs. M2), time-of-day of referring hospital imaging (day : 7:00 to 22:59 *vs* night : 23:00 to 6:59)[1] and intravenous thrombolysis use (yes vs. no) by including the corresponding interaction term into the multivariable mixed logistic regression models. To avoid case deletion in analyses due to missing data on baseline characteristics and outcomes, missing data were imputed by multiple imputations using regression-switching approach (chained equations with m=10). Imputation procedure was performed under the missing at random assumption using all baseline characteristics and study outcomes with a predictive mean matching method for continuous variables and multinomial or binary logistic regression model for categorical variables. Estimates obtained in the different imputed data sets were combined using the Rubin’s rules. Statistical testing was performed at the two-tailed α level of 0.05. Data were analyzed using the SAS software package, release 9.3 (SAS Institute, Cary, NC).

**Supplemental Figure.** Association between inter-hospital transfer time and favorable outcome according to key subgroups


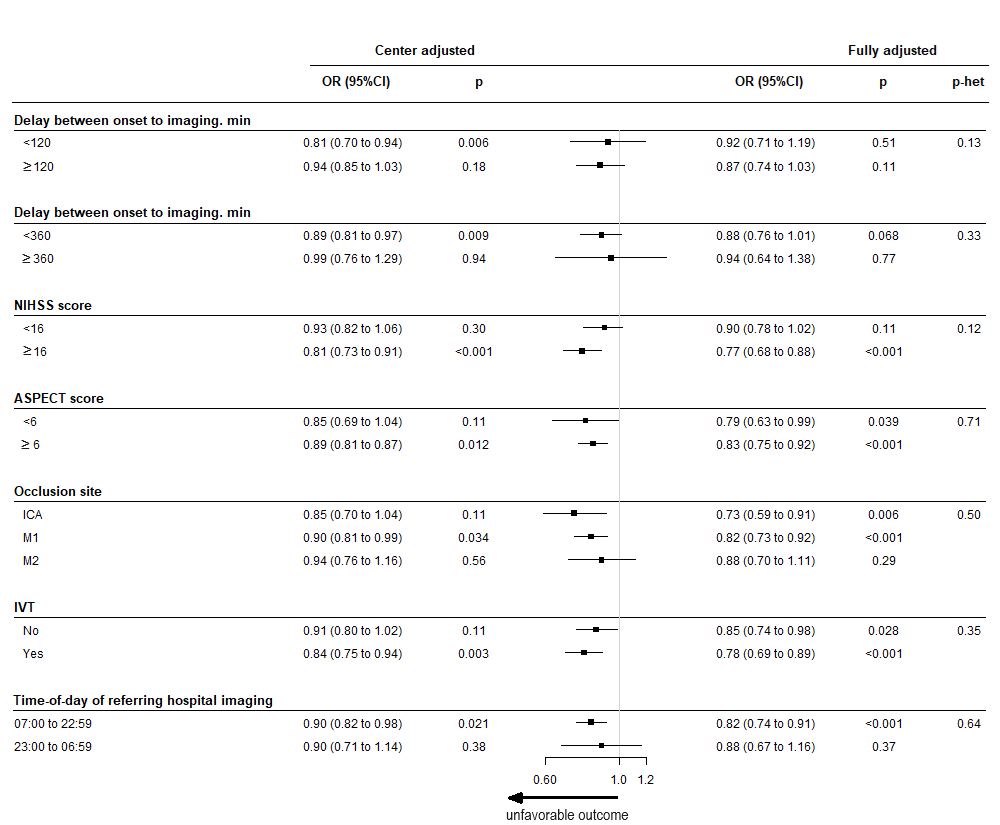


Fully adjusted model adjusted on center, time from symptom onset to referring hospital imaging, age, sex, diabetes, admission (referring hospital) NIHSS and ASPECT scores, admission (referring hospital) occlusion site, intravenous thrombolysis and time-of-day of referring hospital imaging. OR indicates odds ratio per one standard deviation log increase in transfer time obtained using a mixed logistic regression model. P-het indicates P-value for heterogeneity in association of transfer time and favorable outcome according to subgroups calculated by including the corresponding interaction term in multivariable mixed logistic regression models. Values and effects size calculated after handling missing values by multiple imputation.

IVT indicates intravenous thrombolysis.

**References:**

[1]. Saver JL, Klerman EB, Buchan AM*, et al.* Consensus Recommendations for Standardized Data Elements, Scales, and Time Segmentations in Studies of Human Circadian/Diurnal Biology and Stroke. *Stroke*. 2023 **54:** 1943-1949.
